# The Microscopic Anatomy of the Human Body in Health and Disease

**Published:** 1852-02

**Authors:** 


*The Microscopic Anatomy of the Human Body in Health and Disease. Illustrated with numerous drawings in colors.* By Arthur Hill Hassall, M. D., author of a "History of Fresh Water Algæ," Fellow of the Linnæan Society, &c. &c. *With additions to the text and plates, and an introduction containing instructions in Microscopic Manipulations.* By Henry Van-Arsdale, M. D. In two volumes. New York: Pratt, Woodward & Co. 1851.

All who are interested in the study of Microscopic Anatomy, and to whom the above work in its English dress, is doubtless familiar, will be pleased to learn that it has been re-printed in this country, and enriched by the additions of the American editor in the shape of an introduction, and many original American and other drawings. We have watched with great pleasure, the growing interest and zeal which have been of late manifested in this country, in this teeming field, and we refer with pride to the observations of our own micrographers, now beginning to be recognized as authorities all over the world. For many years we have been content to see with foreign eyes and foreign glasses, and to receive as matters of faith, all that has reached us from distant observers. Now we have our own instruments, unexcelled by those of the first English and continental opticians, and our own observers, patient and faithful, upon whom we can rely with confidence for confirmation of all that reaches us. The increasing demand for, and popularity of books upon this and kindred subjects, is the best evidence of the truth of what we have advanced; and in the one before us, we discover what has long been wanting in the English literature of this subject; for the works of Henlé, Lebert, Donné, Robin, Mandl, and others, were "sealed books" to those unfamiliar with the language in which they were written.

One of the main objects of the author of the present volume has been to collect from the various scientific and other journals, the contributions of his own countrymen, and to combine them into a whole, at the same time introducing the labors of other observers, as they served to exemplify the various subjects treated of. Of the American edition we feel that we cannot speak too warmly in praise. It combines all the high scientific characteristics of the original, which are given to us unmutilated, and which render it invaluable to the advanced student in Physiological and Pathological histology, together with an introduction by the Editor, that is admirably adapted to beginners, in which are full descriptions of the different foreign and domestic instruments, and their accessories, the mode of preparation and preservation of objects, together with the fluids used for mounting objects, and the methods of performing this operation; all described in a clear, simple style, that cannot fail to be understood by the merest tyro in microscopy.

It would be a "labor of love" for us to follow the author step by step, through the various departments of the work, did our limits allow, and to exhibit to our readers all that has been done by him and others; but the very nature of the work, in which so much is taught by demonstrative plates, as well as our restricted space, forbid this. We must rest content with the hearty recommendation to all who love the subject, to possess themselves of this admirable work, with the assurance, that their most sanguine expectation will not be disappointed.

One word, as to the "getting up" of the American Edition. It has rarely been our lot to meet a more elegantly executed work, both as regards plates, paper, typography, &c.; it reflects the highest credit upon all concerned. Ten additional plates have been added to the original, together with several wood cuts. The work appears in two volumes, one of which is devoted to the text, and the other to the plates, an arrangment by which the reader is saved the tediousness of turning back to refer to the plates, which must always occur when both are bound together.

